# Evaluation of epithelial mesenchymal transition in patients with chronic obstructive pulmonary disease

**DOI:** 10.1186/1465-9921-12-130

**Published:** 2011-10-05

**Authors:** Sukhwinder S Sohal, David Reid, Amir Soltani, Chris Ward, Steven Weston, H Konrad Muller, Richard Wood-Baker, E Haydn Walters

**Affiliations:** 1 NHMRC National Centre for Research Excellence in Chronic Respiratory Disease, Menzies Research Institute, 17 Liverpool Street, Hobart, 7000, Australia; 2 Applied Immunobiology and Transplantation Research Group, Institute of Cellular Medicine, Newcastle University, William Leech Building Medical School, Newcastle upon Tyne, NE2 4HH, UK

**Keywords:** cytokeratin, clefts, epithelial mesenchymal transition (EMT), inflammatory cells and S100A4

## Abstract

**Background:**

The reticular basement membrane (Rbm) in smokers and especially smokers with COPD is fragmented with "clefts" containing cells staining for the collagenase matrix-metalloproteinase-9 (MMP-9) and fibroblast protein, S100A4. These cells are also present in the basal epithelium. Such changes are likely hallmarks of epithelial mesenchymal transition (EMT). We aimed to confirm the epithelial origin of these Rbm cells, and to exclude potential confounding by infiltrating inflammatory cells.

**Methods:**

Endobronchial biopsy sections from 17 COPD current smokers, with documented Rbm splitting and cellularity were stained for neutrophil elastase (neutrophil marker), CD68 (macrophage/mature fibroblasts), CD4+/CD8+ T lymphocytes, CD19 (B-cells), CD11c (dendritic cells/inflammatory cells), and S100 (Langerhans cells). The number of cells in the Rbm and epithelium staining for these "inflammatory" cell markers were then compared to numbers staining for S100A4, "a documented EMT epitope". Slides were double stained for S100A4 and cytokeratin(s).

**Results:**

In the basal epithelium significantly more cells stained for S100A4 compared to infiltrating macrophages, fibroblasts or immune cells: median, 26 (21.3 - 37.3) versus 0 (0 - 9.6) per mm, p < 0.003. Markedly more S100A4 staining cells were also observed in the Rbm compared to infiltrating macrophages, neutrophils, fibroblasts or immune cells or any sub-type: 58 (37.3 - 92.6) versus 0 (0 - 4.8) cells/mm Rbm, p < 0.003. Cells in the basal epithelium 26 (21.3 - 37.3) per mm) and Rbm (5.9 (2.3 - 13.8) per mm) frequently double stained for both cytokeratin and S100A4.

**Conclusions:**

These data provide additional support for active EMT in COPD airways.

## Introduction

One of the features of chronic inflammatory airway diseases, including COPD, is airway remodelling [1]. In COPD, remodelling may occur as a response to smoking-induced damage to the airways, [2-4] but the details of structural changes and underlying mechanism are poorly described or understood. One potential mechanism contributing to airway fibrosis is transition of airway epithelial cells to a mesenchymal phenotype with myofibroblast characteristics, which then migrate into the lamina propria, a process termed epithelial mesenchymal transition (EMT) [5,6]. There is little knowledge regarding the potential for EMT to occur in COPD airways.

In a recently published study, [7] we provided preliminary data suggesting that EMT is indeed likely in smokers' airways, but is especially active in the airways in current smoker - COPD subjects. These findings were based on 'classic' cell marker staining as used in other publications regarding EMT [8-12]. As a likely hallmark of EMT, we showed that the reticular basement membrane (Rbm) in smokers and COPD is highly fragmented, with elongated spaces or cracks, which we termed "clefts" [7,13], again typical of EMT as described in the literature [10-12,14,15]. These elongated spaces and cracks or clefts in the Rbm were often not empty, but frequently contained cells, which we found were positive for the mesenchymal markers S100A4, vimentin and MMP-9. MMP-9 is a type IV collagenase, which increases in abundance during the process of EMT. Functionally MMP-9 is thought to assist epithelial cell migration by disrupting underlying basement membrane and Rbm and likely to be responsible for the Rbm fragmentation and cleft formation.

Although these changes which we have described are consistent with the presence of active EMT, we need further confirmation; in particular that the Rbm cells we have noted as expressing mesenchymal markers also express epithelial markers. It is also possible that the cell data could be confounded by us staining inflammatory cells infiltrating from below, which could be those positive for S100A4 and MMP-9.

In order to provide further evidence of a potential epithelial origin of cells in the Rbm of smoking COPD subjects, we have undertaken double immunostaining with both an epithelial and mesenchymal marker (cytokeratin-(s) and S100A4, respectively). S100A4 may also be expressed at least weakly by some inflammatory cells [16,17]. We therefore, immunostained biopsies from smoker COPD patients for neutrophils, macrophages, fibroblasts, CD4+ and CD8+ lymphocytes, B cells and also dendritic cells and Langerhans cells.

Since our previous observations had shown that the putative changes of EMT were most marked in actively smoking COPD subjects, this confirmatory "proof-of-concept" study was limited to this most relevant clinical phenotypic group. We have previously studied normal controls and smokers with normal lung function as comparator groups [7], and the latter group also show similar Rbm changes but to a lesser extent., but there seemed little reason to include them in this follow up. Where we focus on where the signal is greatest.

## Methods

### Subjects

In this study stored biopsies were selected from 6 of the seventeen COPD current smokers (COPD-CS) previously recruited using GOLD criteria [18]; all had signed informed consent for ongoing analysis of their biopsies (approved by the Human Research Ethics Committee Tasmania Network). Subjects with a history of other respiratory disorders were excluded. No subjects were receiving corticosteroids (oral or inhaled). These 6 chosen were not exception in any way from the COPD group as a whole, but all had the typical COPD biopsy appearance that we have interpreted as likely airway mucosal EMT.

### Bronchoscopy

Endobronchial biopsies were taken from near sub-segmental carinae of the right lower lobe (using alligator forceps, FB-15C; Olympus, Tokyo) [19].

### Immunostaining

Paraffin embedded sections of 3 μm and 50 μm apart mounted on each slide. At room temperature (RT) sections were stained with the following antibodies: anti-CD11c monoclonal (dendritic cells/inflammatory cells) (Abcam, cat no. ab52632, at 1:20,000 for 2 hours at RT), anti-CD4/CD8 monoclonal antibodies (Novocastra, cat no. NCL-CD4-IF6/cat no. NCL-CD8-4B11, at 1:15 for 2 hours), anti-CD68 monoclonal (macrophages and mature fibroblasts) (Dako, cat no. M0814, at 1:500 for 30 minutes), anti-CD19 monoclonal (B-cells) (Abcam, cat no. Ab31947, at 1:8000 for 2 hours), anti-neutrophil elastase monoclonal (Dako, cat no. Ab31947, at 1:500 for 1 hour), anti-S100 polyclonal (Langerhans cells) (Dako, cat no. Z0311 at 1:3000 for 1 hour) and anti-S100A4 polyclonal (Dako, cat no. A5114, 1:2500 for 90 minutes). Antibodies were visualised using horseradish peroxidase (HRP) conjugated DAKO Envision plus reagent (cat no. K4001, anti-mouse or K4003 anti-rabbit) and diaminobenzidine (DAB) for colour resolution (brown).

Double-staining for cytokeratin and S100A4 was performed using an anti-pan-cytokeratin monoclonal antibody (Dako, cat no. M3515 at 1:50 for 1 hour) and then secondarily bound using Dako "Real" (cat no. K5005) alkaline phosphatase reagent and visualised using Dako (cat no. K0640) liquid permanent red (mayer's haematoxylin was added at this point to elaborate nuclei) this was followed by the anti S100A4 polyclonal antibody (1:2000 for 90 minutes) that was visualised using HRP conjugated DAKO Envision plus (cat no. K4003) reagent and vina green for colour resolution (10 minutes at RT, Biocare, cat no. VG807 H, L). For the negative controls, matched sequential sections were stained with primary antibody replaced with a species-appropriate IgG1 at equivalent dilutions and conditions. We have published using these methods [7,13,19,20].

### Biopsy analysis and quantitation

Computer-assisted image analysis was performed (Leica DM 2500 microscope, Microsystems, Germany), Spot insight 12 digital camera and Image Pro V5.1 (Media Cybernetics, USA) software. For this analysis, we used representative sections (n = 6) from 17 different COPD current smokers for each of the markers of interest. All slides were counted in a single batch by a single experienced observer (SS) with quality assurance on randomly selected slides provided by a professional academic pathologist (HK).

### Statistical analysis

After all counting had been completed, the data were analysed. As data were not normally distributed, results are presented as medians and ranges for cells in the basal epithelium and Rbm stained for each marker respectively, and analysed relative to S100A4 cell staining. Comparisons between different markers were undertaken using the Mann Whitney U test. Associations between variables were assessed using Spearman's rank test. Statistical analyses were performed using SPSS 15.0 for Windows, 2003, with a two-tailed *P*-value ≤ 0.05 being considered significant.

## Results

Demographics of participants are presented in Table 1.

**Table 1:** Demographic details of the subjects who participated in the current study (n = 6), compared to the full original population of patient volunteers from whom they were selected (n = 17)

Group (numbers)	COPD-CS (n = 17)	COPD-CS (n = 6)
GOLD I/GOLD II	10/7	3/3

Male/female	9/8	4/2

Age (Range)	61 (46-78)	59 (55-62)

Smoking pack years (Range)	45 (18-78)	45 (32-70)

FEV1% predicted (Range)†	83 (66-102)	76 (66-102)

FEV1/FVC% (Range)†	59 (46-68)	61 (48-68)

Data expressed as medians and ranges. There were no significant difference between the whole group and the current study subgroups.

† Post BD values after 400μg of inhaled salbutamol

### Confirming epithelial origin of cells stained for S100A4 in the Rbm

**Figure 1:**
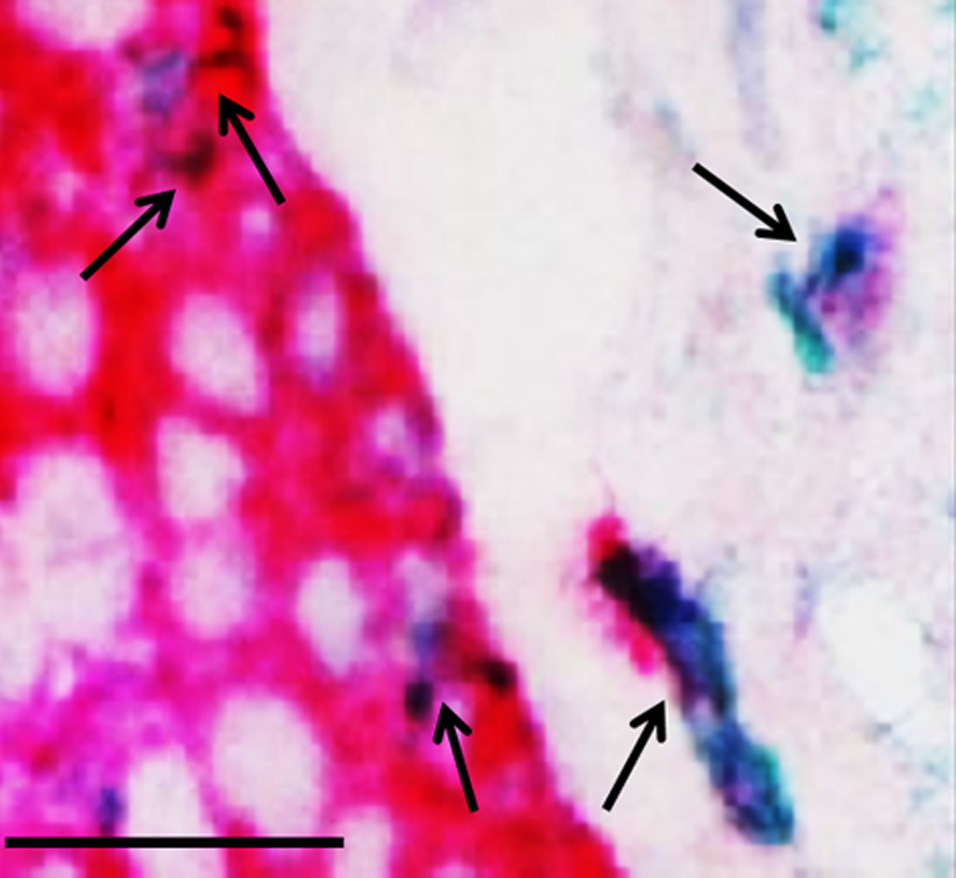
**Bronchial biopsy specimen from a COPD current smoker double-stained for both S100A4 (green) and cytokeratin (red)**. Black arrows showing cells in the basal epithelium and reticular basement membrane (Rbm) and also within the superficial lamina propria, double-stained for both cytokeratin (red, anti-pan-cytokeratin monoclonal antibody, epithelial marker) and S100A4 (green, anti-S100A4 polyclonal antibody, mesenchymal marker). Overall, there are fewer double-stained cells in the Rbm than in the basal epithelium; consistent with loss of epithelial markers as these cells gain mesenchymal markers. Original magnifications 100 ×/1.30 Oil. Scale bar = 50 μm.

To confirm a potential epithelial origin of cells in the Rbm, bronchial biopsy sections were double stained for both cytokeratin-(s) (epithelial marker) and S100A4 (mesenchymal marker). This analysis demonstrated that all cells in the epithelium stained for cytokeratin as might be expected, while about 13.8% of these stained for S100A4; all of the S100A4 cells stained for cytokeratin-(s) (i.e. the cells exhibited both visualization chromogens). In the Rbm, however, fewer cells (about 7%) were positive for cytokeratin-(s) but these cells were all double-positive for both cytokeratin (s) and S100A4 (Figure 1 and Table 2).

**Table 2:** Quantification of S100A4/cytokeratin stained cells and S100A4/cytokeratin double-stained cells in the basal epithelium and Rbm; data expressed as medians and ranges

Markers	No. of basal epithelial cells stained per mm of Rbm	No. of cells staining in Rbm per mm of Rbm
S100A4	26.6 (21.3 - 37.3)	58.1 (37.3 - 92.6)

Cytokeratin-(s)	191.7 (135.3 - 297.6)*	4.3 (0.3 - 11.6)*

Cytokeratin-(s) & S100A4 double staining	26.6 (21.3 - 37.3) (basal epithelial cells +ve for S100A4 as shown above were all +ve for Cytokeratin-(s)	5.9 (2.3 - 13.8)

* Cytokeratin *versus *S100A4, p < 0.003

### Comparative analysis of S100A4 staining with that for immune and inflammatory cells

**Figure 2:**
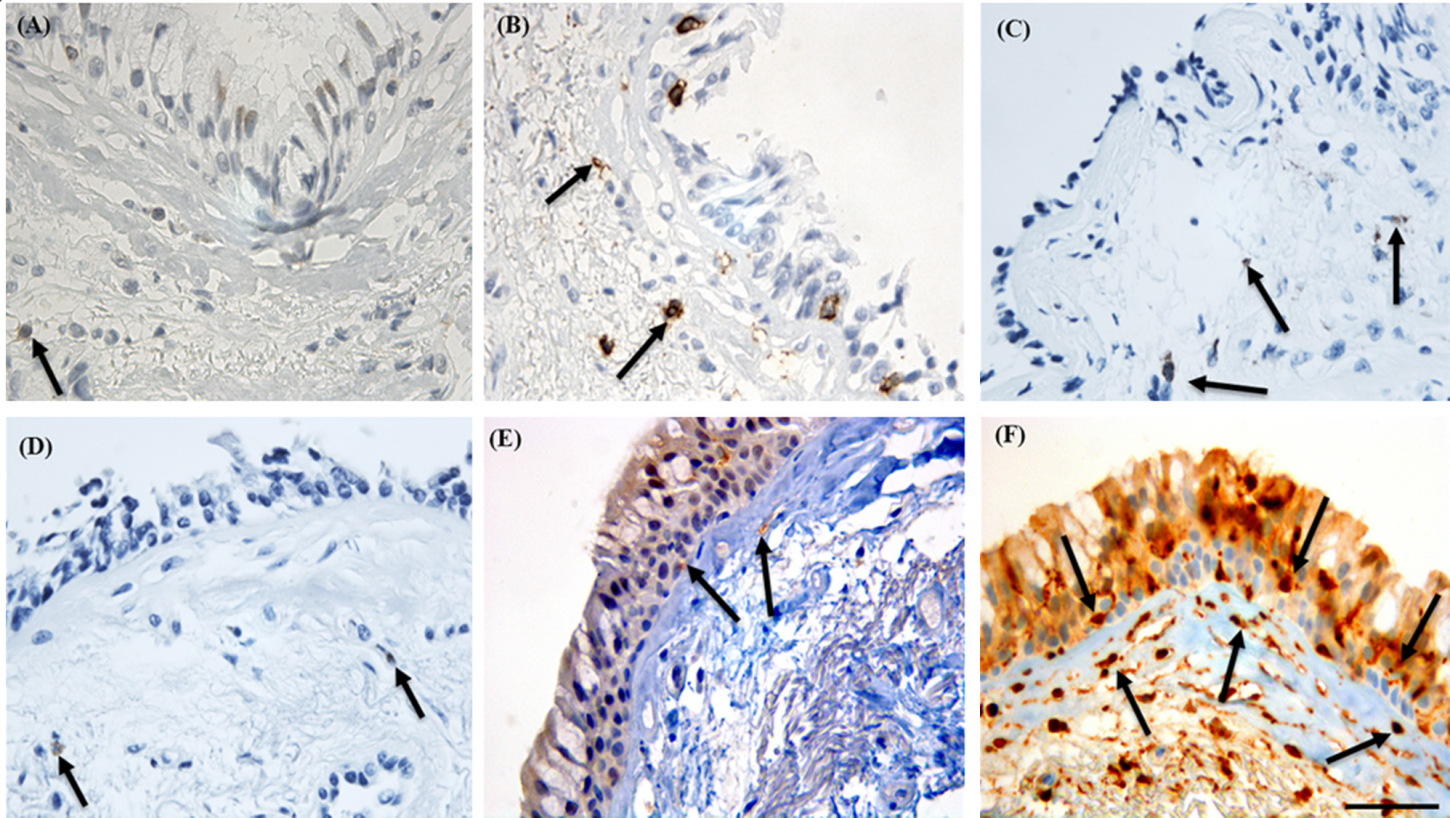
**Bronchial biopsy specimen from a COPD current smoker stained for immune and inflammatory cell markers compared to S100A4**. Black arrows showing cells positive for: **(A) **CD4 (anti-CD4 monoclonal anti-body); **(B) **CD8 (anti-CD8 monoclonal antibody); **(C) **CD68 (anti-CD68 monoclonal antibody; macrophage and mature fibroblast marker); **(D) **neutrophil elastase (anti-neutrophil elastase monoclonal antibody; neutrophil marker); **(E) **CD11c (anti-CD11c monoclonal antibody; dendritic cell/inflammatory cell marker); compared to **(F) **S100A4 (anti-S100A4 polyclonal antibody; mesenchymal marker) stained cells, in the basal epithelium and reticular basement membrane (Rbm). There are many more S100A4 positive cells in the basal epithelium and Rbm compared to cells stained for any inflammatory cell marker. Most of the cells positive for inflammatory cell markers are in the lamina propria below the Rbm. Original magnifications, × 630. Scale bar = 50 μm.

**Figure 3:**
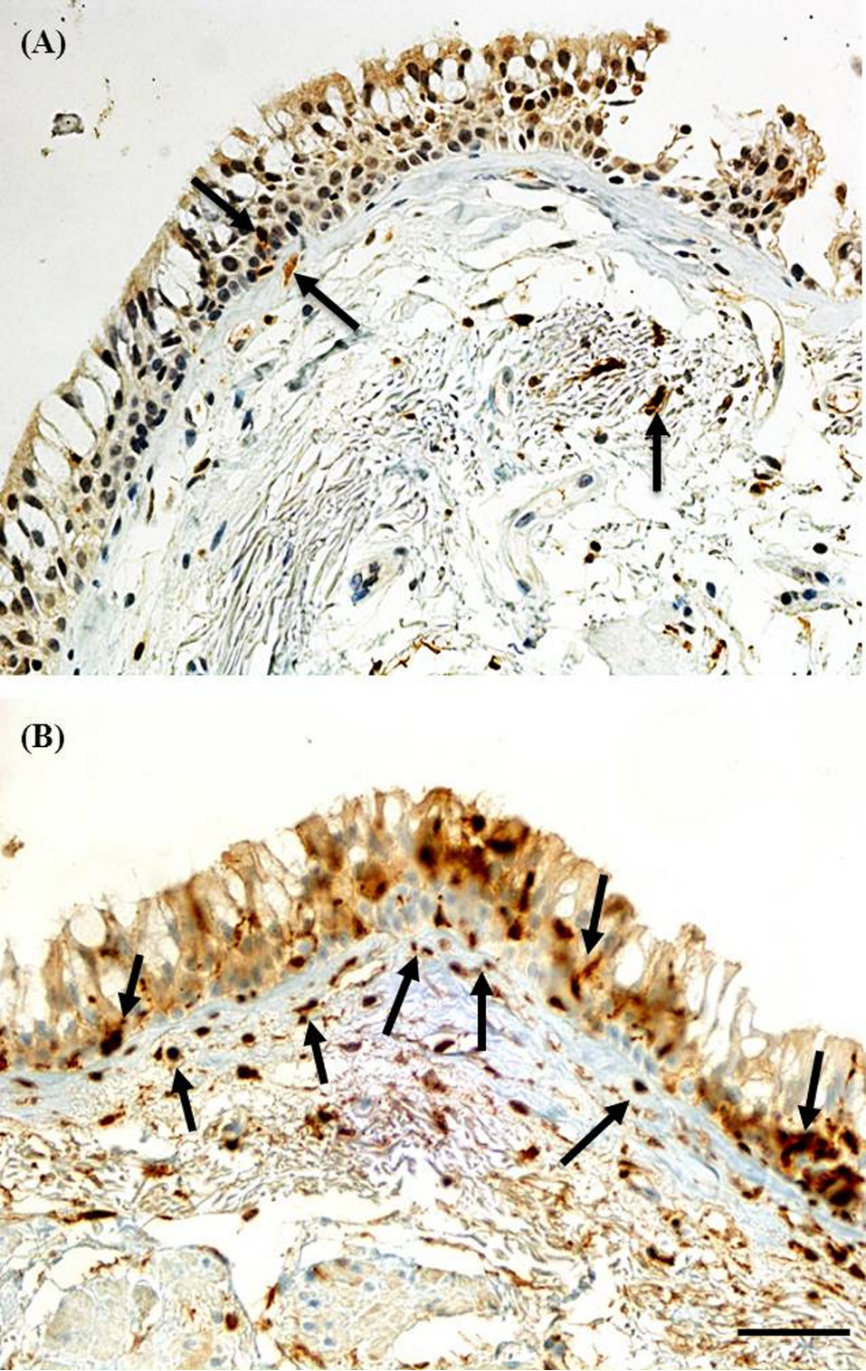
**Bronchial biopsy specimen from a COPD current smoker stained for S100 compared to S100A4**. Black arrows showing cells stained for: **(A) **S100 (anti-S100 polyclonal antibody; Langerhans cell marker); compared to **(B) **S100A4 (anti-S100A4 polyclonal antibody; mesenchymal marker). Original magnifications, × 400. Scale bar = 50 μm.

This comparison demonstrated that there were significantly more (p < 0.003, for all comparisons, Figure 2 and Table 3) cells stained for S100A4 in the basal epithelium and Rbm compared to cells specifically stained for CD4+/CD8+ T lymphocytes, CD19 (B-cells), CD11c (dendritic cell/inflammatory cell marker), S100 (human Langerhans cell marker, Figure 3), neutrophil elastase (neutrophil marker) or CD68 (macrophage and mature fibroblast marker) [21,22].

**Table 3:** Quantification of inflammatory cell markers and S100A4-stained cells in the basal epithelium and Rbm; data expressed as medians and ranges

Markers	No. of basal epithelial cells stained per mm of Rbm	No. of cells staining in Rbm per mm of Rbm
S100A4	26.6 (21.3 - 37.3)	58.1 (37.3 - 92.6)

CD4	0 (0 - 5.8)*	0 (0 - 4.8)*

CD8	5.7 (0.9 - 9.6)*	0.3 (0 - 3.8)*

CD19	0 (0 - 0)*	0 (0 - 0)*

CD11c	1 (0 - 1.8)*	0 (0 - 0.2)*

CD68	0.3 (0 - 2.1)*	0 (0 - 0.6)*

NE	0 (0 - 0)*	0 (0 - 0)*

S100	0 (0 - 1.1)*	0.1 (0 - 2.7)*

* Each marker *versus *S100A4, p < 0.003

## Discussion

We have previously published that EMT could be an active process in human smokers' airways, [7] based on the demonstration of Rbm fragmentation and cleft formation and the presence of cells in both Rbm and basal epithelium staining for established markers of EMT [12,15] such as S100A4 and MMP-9. These changes were present in airway biopsies from asymptomatic smokers with normal lung function, but were most pronounced in currently smoking COPD patients. This confirmatory study, although with limited numbers, focussed on this most relevant clinical phenotypic group, but is likely to be generalizable also to smokers with normal lung function.

Although we have provided rare, human data in human COPD, suggesting the potential for EMT, [7] this conclusion has been open to reviewer scepticism that the markers of EMT that we used, although regarded as 'classic' in the literature, [12,15] were not qualified with further markers, especially showing that the Rbm cells express epithelial markers as well as mesenchymal markers. It is also been suggested that the Rbm cell data could be confounded by infiltration from below by inflammatory cells, which could at least potentially be positive for S100A4 and MMP-9. Our COPD subjects in this study were on as needed "relief" bronchodilator medication only, and it is unlikely that the discussed appearances are due to confounding by therapy, which we have deliberately tried to avoid as much as possible.

In the present study we have followed up our previous findings by demonstrating that cells in the Rbm positive for mesenchymal markers did frequently stain with an epithelial marker. Further, the present study illustrates that there are cells in the Rbm that double stain for cytokeratin-(s) and the "EMT marker" S100A4, representing further evidence of EMT in smoking-related COPD. Furthermore, our staining of tissue comprehensively for markers of a variety of inflammatory cells indicates that our data were not confounded by significant infiltration of the Rbm by inflammatory or immune cells.

Although only a relatively small number of cells in the Rbm could be demonstrated to stain with cytokeratin, it is possible that more (indeed most) of these Rbm cells are actually of epithelial origin, as it is well documented that during development of EMT, epithelial cells lose epithelial markers as they gain mesenchymal markers. Therefore, a cell which has gone through a complete transition will not express epithelial markers. It is, however, reassuring from the point of view of our hypothesis about active EMT in the airways in smokers and COPD, that we have been able to demonstrate at least a reasonable number of double-stained cells in the Rbm. Similarly, the frequent presence of cells in the basal epithelium staining for S100A4 is further evidence of EMT in these COPD airway biopsies. The data are consistent with a model where migratory cells gain the S100A4 mesenchymal marker early, but then rapidly loose epithelial markers as they migrate through the Rbm.

We found that there were very few CD8+ and CD4+ T lymphocytes and no CD19 B-cells in the Rbm and basal epithelium. Cells positive for CD19 were mainly in the lamina propria and hardly detectable in the basal epithelium or Rbm. We also confirmed that our Rbm cellular S100A4 staining was not confounded by infiltrating dendritic cells or Langerhans cells for which sections were stained for CD11c (dendritic cell/inflammatory cell marker) [23] and S100 (human Langerhans cell marker) [24]. Although they were certainly present, there were relatively few such cells in the basal epithelium and Rbm compared to S100A4-stained cells. Most of the cells positive for the inflammatory cells studied were observed in the lamina propria below the Rbm, and not in the Rbm itself. EMT is a process defined by loss of epithelial markers and gain of mesenchymal markers. A function of this is that there is no single definitive marker for EMT. S100A4 is widely published as representing a reasonable marker for EMT [8-12]. We have also shown that these S100A4 positive cells in the Rbm also double-stain for another mesenchymal protein, vimentin, [7] widely used as an EMT marker [12,15].

EMT has only recently been recognised in the human lung or airway, [6,8] and now we provide further confirmatory evidence for its active presence in COPD airways [7]. However, EMT is well described in lung embryogenesis, [25] metastatic malignant disease [26] and as part of the repair process in renal disease following tissue injury [27]. Active EMT is indicated by the degradation of underlying epithelial basement membrane and transition of epithelial cells into mesenchymal cells with migratory potential, such that they move away from the epithelium in which they originated into deeper tissue [12,15].

Active airway EMT in COPD could potentially relate to subsequent fibrotic activity in the sub-epithelial tissue, or just reflect severity of COPD pathology. COPD is a complex disease with physiologically a mixture of a variable emphysema component and intrinsic airway narrowing. Pathologically the airways are always abnormal and it is this component we have sampled from large airway biopsies. Airway obstruction is thought to be mainly in the small airways, but the pathology affects the whole bronchial tree [2-5]. There are in fact very few data available on fibrosis or alterations in fibroblast/fibrocyte populations in the airway in COPD. Our data taken together suggest that remodelling changes can be more exaggerated in established COPD and especially in current smokers [28,29]. This area of pathology needs more specific study and remodelling changes need relating to changes in airway calibre and stiffness. Even if there are no immediate physiological consequences, there may well be implication for other COPD-associated processes and especially airway malignancy where the concurrence of EMT may be a significant part of tumour pathogenesis.

EMT has been described in metastatic malignant disease especially when associated with S100A4 expression. Epithelial cell nuclear expression of S100A4 strongly correlates with both active EMT and metastatic disease in the oncology literature [8,30,31]. Increased expressions of S100A4 and MMP-9 are observed in human non-small cell lung cancer (NSCLC) and have significant correlations with clinical and biological behaviour of such cancer cells [8,30,31]. Epidermal growth factor receptor (EGFR) is also over-expressed in many types of cancers, including NSCLC and we have shown that this is co-expressed in the epithelium as part of the whole 'COPD phenotype signal' [7]. It has been shown that clinical responsiveness to EGFR inhibition using the "biologic" erlotinib is directly linked to the degree of EMT in NSCLC [28]. Our findings of smoking-related active EMT may help understanding of why lung cancer is so common in smokers, but especially so in those with COPD where we have found EMT to be most marked.

Our study is of course limited by the fact that it was carried out in living human subjects and is based on 'static' pictures. Further study may require COPD model systems in animals and/or human cell culture. This might allow direct, vital imaging to observe dynamic real-time changes in cell phenotype and cellular migration during EMT.

It has been argued that Rbm fragmentation in smokers and COPD biopsies in our studies represents an "artefact" due to tissue processing. Against this is the fact that clefts contained cells, and also, to some extent vessels [13]. Further, tissues from normal healthy controls [7] and asthmatics (data not shown) were processed in exactly the same way, but rarely showed any such Rbm fragmentation.

## Conclusions

In summary, double staining of cells by both epithelial and mesenchymal markers in both the basal layer of the epithelium and within the fragmented Rbm is supportive of our hypothesis that EMT is indeed likely to be an active process in the airways in smokers, and especially those of current-smoking COPD patients. We also provided evidence that these S100A4 stained cells are not being confused with infiltrating inflammatory or immune cells. We conclude that the most likely origin of the S100A4 and cytokeratin dual positive cells apparent within the Rbm in COPD is from the airway epithelium, with this representing additional evidence that EMT may occur.

## Competing interests

Sukhwinder S. Sohal, David Reid, Amir Soltani, Chris Ward, Steven Weston, H. Konrad Muller, Richard Wood-Baker and E. Haydn Walters declare that they have no competing interests.

## Authors' contributions

**SSS: **performed the histological, statistical analysis and writing of paper.

**DR: **performed bronchoscopies and clinical assessments, and contributed to writing of the paper.

**AS: **assisted in histological analyses.

**CW: **advised on EMT science and histology.

**SW: **performed the tissue processing and immunostaining of samples.

**HKM: **advised on histology strategy and quality control.

**RWB: **performed bronchoscopy and clinical assessments.

**EHW: **design of study, clinical assessments, overview of all analyses and writing of paper. All authors have read and approved the final manuscript.
